# Synergistic improvement in spring maize yield and quality with micro/nanobubbles water oxygation

**DOI:** 10.1038/s41598-019-41617-z

**Published:** 2019-03-26

**Authors:** Yunpeng Zhou, Yunkai Li, Xiujuan Liu, Keyuan Wang, Tahir Muhammad

**Affiliations:** 1 College of Water Resources and Civil Engineering, China Agricultural University, Beijing, 100083 China; 2 Fujian Provincial Investigation, Design & Research Institute of Water Conservancy & Hydropower, Fuzhou, 350001 China

## Abstract

Soil oxygen shortages in root areas have negative effects on crop growth and decrease crops yield and quality, and soil hypoxia conditions will be aggravated by application of subsurface drip irrigation (SDI). A two-year field experiment was conducted to evaluate the response of maize to micro/nanobubbles oxygation (MNBO) at three dissolved oxygen (DO) concentrations (10, 20 and 30 mg/L) and seven MNBO periods (vegetative stage, reproductive stage, filling and ripening stage, combination of two stages and the whole growth stage) in addition to a control treatment (CK, no oxygation during the growth period). Our results revealed that the MNBO treatments increased maize root dry weight, root length density and root surface area in 0–20 cm soil. The highest yield was obtained in O_20_A (MNBO at 20 mg/L DO during the growth period), with an increase of 11.66% relative to CK. Crude ash, starch and vitamin C were improved by application of MNBO at 20 mg/L DO. However, excessive oxygen adversely affected maize growth, decreasing the maize yield in 2013 relative to CK. The results suggest that application of MNBO at 20 mg/L DO during the growth period of spring maize was appropriate.

## Introduction

Subsurface drip irrigation (SDI), generally defined as the application of water below the soil surface through emitters with discharge rates similar to those of drip irrigation, is an efficient water-saving irrigation method which was developed to improve drip irrigation technology^1^. SDI can maintain the surface soil dry, effectively prevent and control the overgrowth of weeds; reduce surface evaporation, surface runoff and deep percolation; improve irrigation water use efficiency (IWUE); promote crop growth; increase crop yield, quality and fertilizer utilization; reduce the application of pesticides and use unconventional water sources for irrigation to ease the pressure of water shortages^2–6^.

However, SDI eliminates air from the pores of the root zone while providing moisture and nutrients to crops, resulting in temporary or long-term anaerobic environment in the root zone^7^. The diffusion rate of the dissolved oxygen (DO) in water into the deep layers of the soil is significantly reduced under high soil moisture, leading to a reduction in soil oxygen content^8^. When the oxygen content in the soil is insufficient, one of the earliest stress responses of plants is stoma closure^9^, which in turn adversely affects crop photosynthesis^10^. When hypoxia stress occurs, the relative transpiration of crops is immediately greatly reduced. Persistent hypoxia can have a number of adverse effects on crops, such as nutrient deficiency, metabolic inhibitor generation and increased incidence of root diseases^11^. When plants are exposed to hypoxia stress, growth is weakened, new leaf formation is blocked, the number of leaves and leaf area decrease, leaves quickly turn yellow, wilting occurs, the dry matter content decreases, and fruit quality is poor^12–14^.

Oxygation through the full pipe system of SDI delivers the moisture and oxygen required for crop growth to the root zone, which effectively improves the insufficient soil aeration and the oxygen environment in crop root zone, consequently promoting crop growth, yield and quality. Previous studies used air compressors or air injectors as forced air intake systems to transport air to the crop root zone through a buried pipeline^15–17^. Forced air intake can relieve the hypoxia of the rhizosphere, increase root dry weight and activity, and enhance the absorption of water by the roots^18,19^. It has been verified that oxygation could be used to alleviate soil hypoxia and increase crop yield and quality. When the soil moisture content is high, the forced air intake method plays a positive role in promoting the crop yield. However, when the soil moisture content is low, this method has adverse effects on crop yield^20^. Most current oxygation methods focus on adding hydrogen peroxide to irrigation water and the use of venturi injectors or air syringes^7,21,22^. The spread of the air delivered into soil through SDI system emitters is asymmetrical in all directions. The air most likely diffuses into the atmosphere through several preferred channels; this is called the “chimney affect”^23^, and results in a low gas utilization rate and a short contact time with the crop roots.

The size of micro/nanobubbles (MNBs) is between that of microbubbles and nanobubbles^24–26^. MNBs can be generated by ultrasonic cavitation, chemical reactions, electrolysis and other methods^27–29^. MNBs have some unique properties, including stability, persistence, large specific surface area, slow rise in water, and good mass transfer coefficient. Broken MNBs can generate shock waves and local high temperatures^30–32^, and thus have important applications in the metallurgical and environmental fields. Therefore, the combination of MNBs and oxygation is expected to solve the aforementioned problems. It was hypothesized that MNB oxygation (MNBO) through an SDI system could promote crop growth and root development and, improve the yield and nutritional quality of spring maize. The objectives of this paper were to: 1) investigate the effects of different MNBO periods and DO concentration on the growth, yield, IWUE and nutritional quality of spring maize; 2) reveal how MNBO regulates maize yield and quality; and 3) determine the optimal application pattern of MNBO for spring maize.

## Results

### Maize height and stem diameter

Measurements of maize height and stem diameter were collected every 15 days from the beginning of the experiment until harvest in 2013 (Table 1). Analysis of variance (ANOVA) results indicated that MNBO stage had no significant effect on maize height except for at 30 days and 105 days. The maize stem diameter significantly differed between different MNBO treatments from 30 days to 105 days (*p* < 0.05), increasing by 7.01–24.77%, 6.82–26.89%, 8.41–27.83% and 8.44–29.55% compared to CK for 30, 45, 60 and 75 days, respectively. ANOVA results indicated that maize height and stem diameter significantly increased at 60 days by 5.86–7.04% and 12.99–18.83%, respectively. There were no significant differences in maize height and stem diameter after 60 days in the DO treatments.

**Table 1:** Maize height and stem diameter of spring maize in 2013.

Treatment	15d		30d		45d		60d		75d		90d		105d	
	Maize height	Stem diameter	Maize height	Stem diameter	Maize height	Stem diameter	Maize height	Stem diameter	Maize height	Stem diameter	Maize height	Stem diameter	Maize height	Stem diameter
O_20_V	28.9 ± 1.4a	0.9 ± 0.0a	88.3 ± 1.2a	25.9 ± 1.5a	157.7 ± 4.5a	33.5 ± 0.7a	243.9 ± 2.0ab	39.5 ± 0.9a	272.7 ± 3.2a	39.9 ± 0.8a	283.7 ± 5.0ab	35.3 ± 2.5a	287.3 ± 4.5ab	35.2 ± 0.7a
O_20_R	26.8 ± 2.4a	0.9 ± 0.1a	85.6 ± 7.2abc	26.7 ± 0.9a	159.8 ± 6.2a	32.0 ± 0.9ab	243.2 ± 1.4ab	37.3 ± 0.9ab	268.6 ± 1.4a	36.9 ± 0.9b	275.1 ± 8.1bc	31.4 ± 0.9bd	275.3 ± 8.5bc	28.8 ± 1.7bc
O_20_F	27.1 ± 1.0a	0.9 ± 0.1a	80.3 ± 1.0 cd	25.2 ± 1.3ab	152.6 ± 3.3ab	30.5 ± 1.3bcd	240.5 ± 5.3ab	36.8 ± 1.3bc	270.9 ± 5.6a	36.1 ± 1.2bc	271.4 ± 11.8bc	29.9 ± 1.3bcde	272.3 ± 10.2 cd	27.4 ± 1.7bc
O_20_VR	29.0 ± 2.2a	1.0 ± 0.0a	82.3 ± 2.1bcd	22.9 ± 1.3b	155.4 ± 3.2a	28.2 ± 1.3ef	239.6 ± 2.0ab	33.5 ± 1.3d	270.0 ± 2.2a	33.4 ± 1.4d	271.5 ± 14.9bc	27.6 ± 1.3e	271.8 ± 14.7 cd	27.0 ± 0.3c
O_20_VF	28.1 ± 1.5a	0.9 ± 0.1a	78.9 ± 1.0 cd	23.4 ± 0.8b	155.8 ± 1.2a	28.5 ± 0.7def	237.6 ± 5.8b	34.9 ± 0.7 cd	271.0 ± 5.8a	33.9 ± 0.8 cd	278.4 ± 6.6ab	28.2 ± 0.5e	278.9 ± 6.6abc	28.1 ± 1.6bc
O_20_RF	29.2 ± 0.3a	1.0 ± 0.1a	88.4 ± 2.1a	26.0 ± 1.5a	156.7 ± 2.8a	31.0 ± 1.5bd	240.8 ± 2.6ab	37.5 ± 1.5ab	270.2 ± 2.6a	35.3 ± 2.1bcd	283.0 ± 7.6ab	30.7 ± 2.2bcd	283.3 ± 7.6abc	28.0 ± 3.0bc
O_10_A	26.6 ± 1.5a	0.9 ± 0.1a	79.9 ± 3.6 cd	23.0 ± 0.4b	159.5 ± 3.7a	29.0 ± 0.4 cde	242.2 ± 2.7ab	34.8 ± 0.4cd	272.6 ± 2.7a	34.8 ± 0.5 cd	274.1 ± 7.3bc	30.6 ± 2.2bcde	274.7 ± 7.3bcd	29.7 ± 2.7bc
O_20_A	28.9 ± 1.9a	0.9 ± 0.1a	84.4 ± 3.4abc	26.2 ± 1.1a	156.7 ± 2.4a	31.7 ± 1.1ab	244.9 ± 3.0a	37.7 ± 1.5ab	274.8 ± 3.0a	36.6 ± 1.6b	290.5 ± 1.4a	31.4 ± 1.1bc	290.7 ± 1.4a	28.8 ± 1.8bc
O_30_A	29.0 ± 1.7a	1.0 ± 0.1a	87.6 ± 0.9ab	25.8 ± 1.2a	157.8 ± 3.3a	30.9 ± 1.2bc	243.4 ± 2.9ab	36.7 ± 1.4bc	272.8 ± 2.7a	36.2 ± 1.3bc	277.3 ± 3.4ab	31.8 ± 2.1b	277.8 ± 3.4abc	30.6 ± 1.6b
CK	26.5 ± 0.8a	0.9 ± 0.0a	77.7 ± 2.3d	21.4 ± 2.1b	145.9 ± 4.3d	26.4 ± 2.1 f	228.8 ± 5.3c	30.9 ± 2.3e	256.2 ± 4.0b	30.8 ± 2.4e	260.0 ± 5.7c	28.5 ± 0.7cde	260.7 ± 5.7d	28.0 ± 0.6bc
Significant level														
MNBO Stages	ns	ns	*	*	ns	**	ns	**	ns	**	ns	**	*	**
Dissolve oxygen	ns	ns	*	*	ns	*	*	*	ns	ns	ns	ns	ns	ns

Notes: Maize height in cm and stem diameter in mm. Data were shown in mean ± standard deviation (n = 15). O_10_, O_20_, O_30_ represent dissolved oxygen concentration of 10, 20 and 30 mg/L. V, R, F, A represent micro/nanobubbles oxygation (MNBO) at the vegetative stage, the reproductive stage, the filling and ripening stage and the whole growth period, respectively. VR, VF, FR represent combination of V and R, V and F, R and F. 15d, 30d, 45d, etc. were the date of measurements. Data followed by different lowercase letters in the same column indicate statistically significant differences. ns, no significance; *p < 0.05; **p < 0.01.

### Root dry weight (RDW), root length density (RLD) and root surface area (RSA)

**Figure 1:**
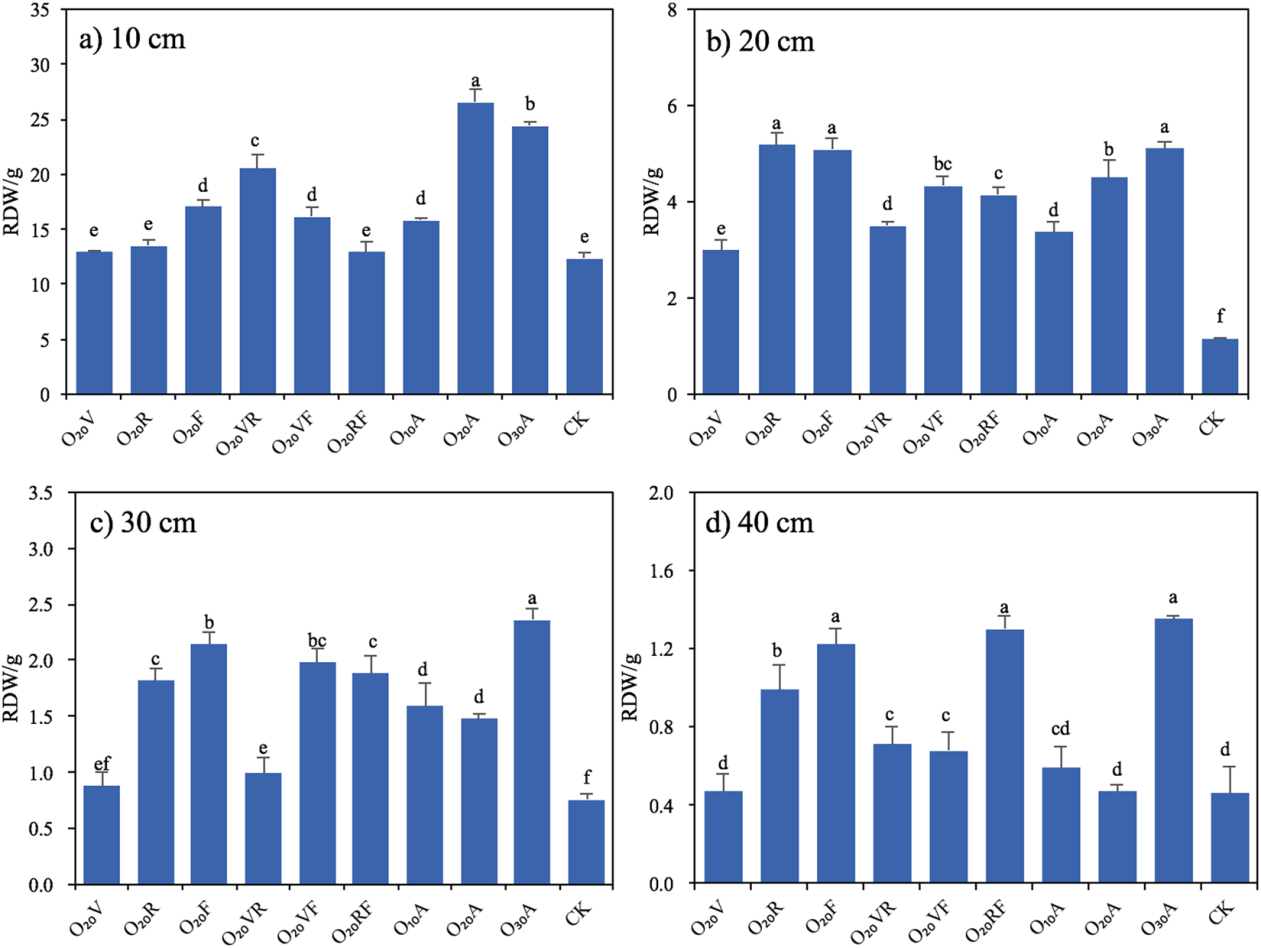
Maize root dry matter (RDW) in the soil layer of (**a**) 10 cm, (**b**) 20 cm, (**c**) 30 cm and (**d**) 40 cm in 2013. Notes: Data were shown in mean ± standard deviation (n = 5). Values without the same letters within the same column at each site are significantly different (*p* < 0.05, LSD’s test).

Most of the maize root system is concentrated in the top 0.4 m soil layer^6,33^. The vertical distribution of RDW (Fig. 1) in the different treatments was similar, mostly concentrated in the 0–10 cm soil layer (Fig. 1a), which accounted for 58.14–80.14% of the total RDW. The RDW in the soil below 10 cm decreased with increasing soil depth (Fig. 1b–d), and below 40 cm, there was little difference between treatments. MNBO significantly improved maize RDW (*p* < 0.05) in the 0–40 cm soil layer. The effect of MNBO on RDW was most pronounced in the 10–20 cm soil layer (Fig. 1b), in which RDW increased by 0.55–2.36 times compared with CK. The vertical distribution of maize RDW in the 0–10 cm soil layer showed an initial increase followed by a decrease with increasing DO concentration. RDW_O20A_, was the highest, at 26.55 g, and increased by 68.04% and 8.59% relative to RDW_O10A_ and RDW_O30A_, respectively. Moreover, the vertical distribution of maize RDW in the 0–10 cm soil layer revealed that the longer the duration of MNBO was, the more pronounced the promotion of RDW. Compared to MNBO at individual growth stages (O_20_V, O_20_R, O_20_F) and combination of two growth stages (O_20_VR, O_20_VF, O_20_RF), O_20_A increased RDW by 29.26–105.18%. There was no significant difference in RDW between O_20_V, O_20_F, and O_20_R in the 0–10 cm soil layer.

**Figure 2:**
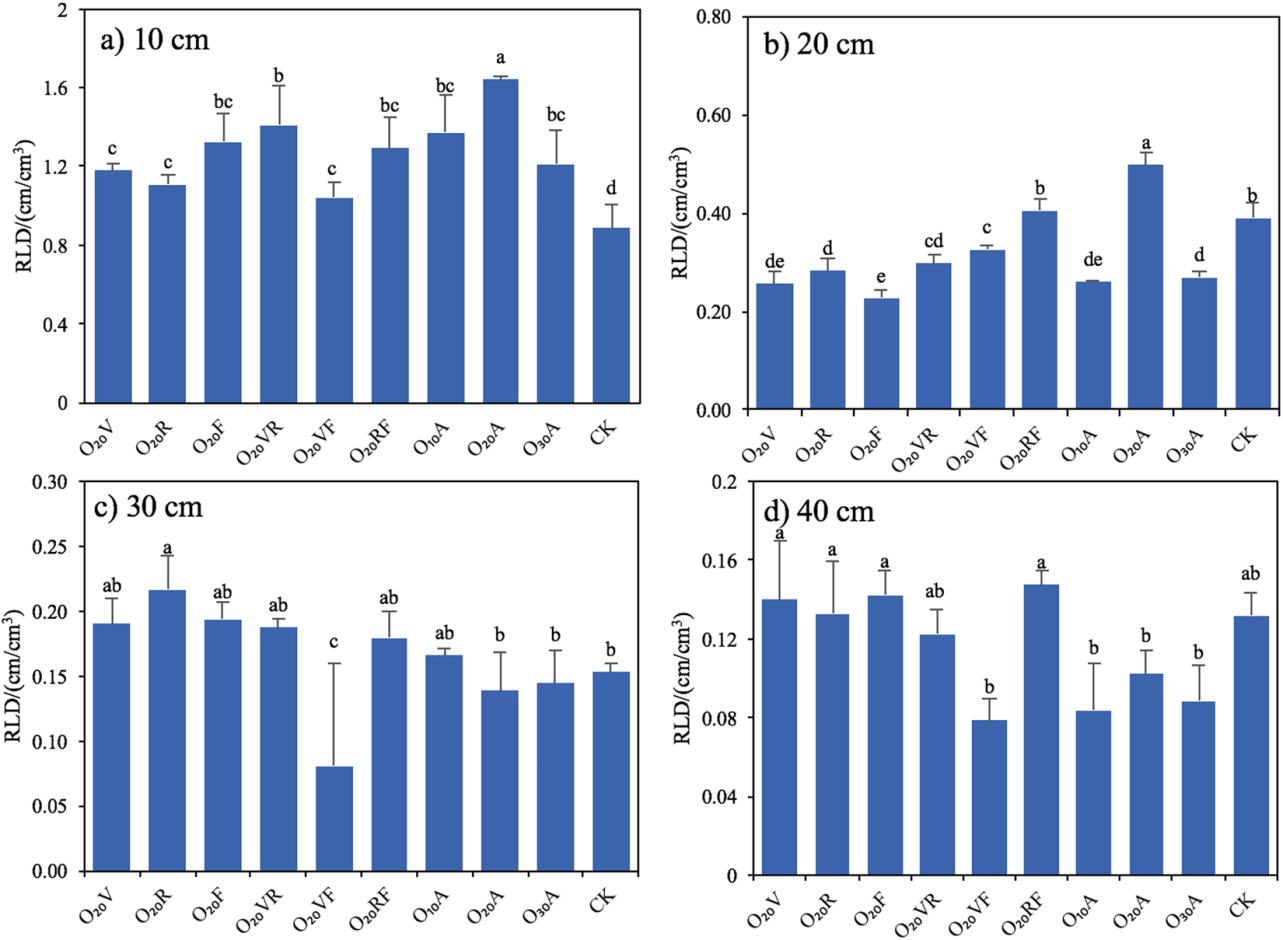
Maize root length density (RLD) in the soil layer of (**a**) 10 cm, (**b**) 20 cm, (**c**) 30 cm and (**d**) 40 cm in 2013. Notes: Data were shown in mean ± standard deviation (n = 5). Values without the same letters within the same column at each site are significantly different (*p* < 0.05, LSD’s test).

The effects of MNBO period on RLD **(**Fig. 2**)** distribution indicated that the longer the MNBO duration was, the more concentrated the roots in the shallow soil layer. Compared to CK, MNBO treatments increased maize RLD by 2.14–47.64%. It was inferred that MNBO period had a significant effect on RLD in the 0–40 cm soil layer (*p* < 0.05) and DO concentration affected RLD in the 0–30 cm soil layer. The highest RLD in the soil layer of 0–40 cm was observed in O_20_A and accounted for 93.34% of the total RLD.

**Figure 3:**
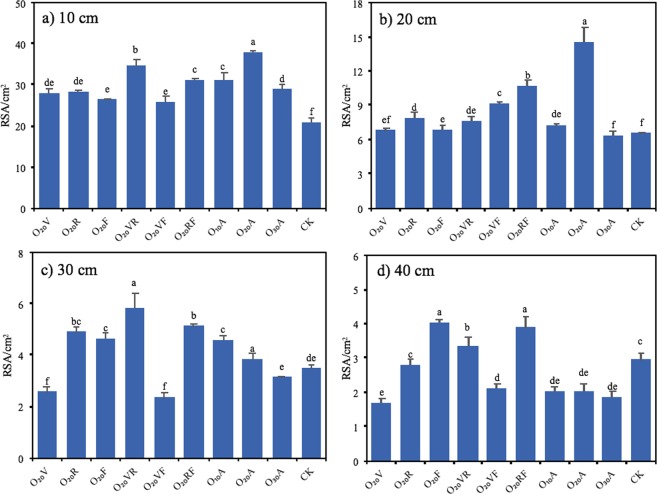
Maize root surface area (RSA) in the soil layer of (**a**) 10 cm, (**b**) 20 cm, (**c**) 30 cm and (**d**) 40 cm in 2013. Notes: Data were shown in mean ± standard deviation (n = 5). Values without the same letters within the same column at each site are significantly different (*p* < 0.05, LSD’s test).

The larger the total RSA **(**Fig. 3**)** of the maize roots was, the larger the area from which roots could absorb nutrients and moisture from the soil, and the better growth ability of maize. MNBO remarkably impacted maize RSA in the 0–20 cm soil layer (Fig. 3a,b), but below 20 cm (Fig. 3c,d), the differences between treatments were not obvious. The vertical distribution of maize RSA at different DO concentrations in the 0–20 cm soil layer revealed that RSA_O20A_ was the largest in the 0–10 cm and 10–20 cm soil layers, followed by RSA_O10A_ and RSA_O30A_, with respective declines of 21.70% and, 31.34% in the 0–10 cm soil layer and 100.00% and, 129.70% in the 10–20 cm soil layer compared with O_20_A.

### Yield, irrigation water use efficiency (IWUE), rainfall use efficiency (RUE) and water use efficiency (WUE)

The maize yield, IWUE, RUE and WUE had statistically differences in 2013 and 2014 (Table 2). Application of MNBO significantly increased the maize yield except for O_30_A in 2013, in which maize yield decreased 10.97%. As DO concentration increased, spring maize yield first increased and then decreased. The O_20_A treatment resulted in a yield that was 7.02% and 8.17% greater than the yields in the O_10_A and O_30_A treatments, respectively. Compared with CK, an increase of 11.66% was observed in O_20_A. The maize yields differed significantly among different MNBO stages. Generally, the results showed that the longer the oxygation duration was, the higher the maize yield. O_20_A produced more maize grain than did MNBO application at in each pair of growth stages (O_20_VR, O_20_VF, O_20_RF) and MNBO application at a single growth stage (O_20_V, O_20_F, O_20_R). The O_20_A treatment increased the maize yield by 3.37–11.11% relative to the others treatments. The IWUE, PUE and WUE were generally increased compared to CK except for O_30_A, which decreased the maize yield. The IWUE, RUE and WUE were determined by maize yield, irrigation quota, rainfall per hectare, and total water consumption. The trends of IWUE, RUE and WUE were consistent with maize yield due to the irrigation quota, rainfall conditions and total water consumption being the same.

**Table 2:** Maize yield, irrigation water use efficiency (IWUE), rainfall use efficiency (RUE) and water use efficiency (WUE) under different treatments in 2013 and 2014.

Treatment	2013				2014			
	Yield	IWUE	RUE	WUE	Yield	IWUE	RUE	WUE
O_20_V	11.82 ± 0.31b	4.14 ± 0.3b	5.84 ± 0.0b	2.41 ± 0.09b	12.03 ± 0.78b	4.44 ± 0.29b	3.45 ± 0.22b	1.94 ± 0.13b
O_20_R	11.98 ± 0.23ab	4.20 ± 0.2ab	5.89 ± 0.2ab	2.45 ± 0.10ab	11.88 ± 1.01b	4.39 ± 0.37b	3.41 ± 0.29b	1.92 ± 0.16b
O_20_F	11.94 ± 0.82ab	4.18 ± 0.8ab	5.86 ± 0.0ab	2.44 ± 0.17ab	12.39 ± 1.19ab	4.57 ± 0.44ab	3.55 ± 0.34ab	2.00 ± 0.19ab
O_20_VR	12.01 ± 0.34ab	4.20 ± 0.2ab	5.89 ± 0.11ab	2.45 ± 0.05ab	11.96 ± 1.26b	4.41 ± 0.47b	3.43 ± 0.36b	1.93 ± 0.20b
O_20_VF	12.17 ± 0.84ab	4.28 ± 0.8ab	5.98 ± 0.8ab	2.49 ± 0.21ab	12.14 ± 1.03b	4.48 ± 0.38b	3.48 ± 0.3b	1.96 ± 0.17b
O_20_RF	12.18 ± 0.23ab	4.29 ± 0.2ab	5.99 ± 0.2ab	2.49 ± 0.07ab	12.38 ± 0.44ab	4.57 ± 0.16ab	3.55 ± 0.13ab	2.00 ± 0.07ab
O_10_A	12.07 ± 0.82ab	4.24 ± 0.8ab	5.94 ± 0.0ab	2.47 ± 0.17ab	12.03 ± 0.85b	4.44 ± 0.31b	3.45 ± 0.24b	1.94 ± 0.14b
O_20_A	12.59 ± 0.46a	4.42 ± 0.4a	6.19 ± 0.4a	2.57 ± 0.05a	13.20 ± 0.54a	4.87 ± 0.2a	3.79 ± 0.16a	2.13 ± 0.09a
O_30_A	10.12 ± 0.59c	3.54 ± 0.5c	4.97 ± 0.5c	2.07 ± 0.12c	11.93 ± 1.15b	4.40 ± 0.4b	3.42 ± 0.33b	1.93 ± 0.19b
CK	11.23 ± 0.50b	3.93 ± 0.5b	5.51 ± 0.5b	2.29 ± 0.06b	11.87 ± 0.76b	4.38 ± 0.28b	3.41 ± 0.22b	1.92 ± 0.12b
Significant level								
MNBO stage	*	*	*	*	*	*	*	*
Dissolve oxygen	*	*	*	*	**	**	**	**

Notes: Yield in 10^3^ kg/ha, IWUE, RUE and WUE in kg/m³. MNBO was the abbreviation of micro/nanobubbles oxygation. Data were shown in mean ± standard deviation (n = 15). Values without the same letters within the same column at each site are significantly different (*p* < 0.05, LSD’s test). ns, no significance; *p < 0.05; **p < 0.01.

### Nutritional quality

**Figure 4:**
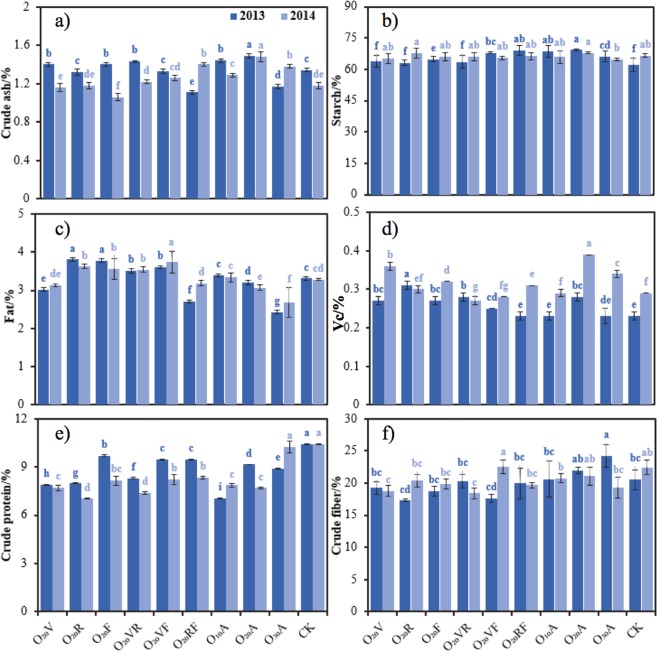
Average values of parameters to maize (**a**) crude ash, (**b**) starch, (**c**) fat, (**d**) vitamin C, (**e**) crude protein and (**f**) crude fiber in 2013 and 2014. Notes: Crude ash, fat and starch in g 100/g, crude protein, vitamin C and crude fiber in %. Data were shown in mean ± standard deviation (n = 5). Values without the same letters within the same column at each site are significantly different (*p* < 0.05, LSD’s test).

The maize nutritional quality data are summarized in Fig. 4. According to the ANOVA of two years of data, the content of maize crude ash (Fig. 4a), fat (Fig. 4c), vitamin C (Fig. 4d), crude protein (Fig. 4e) and crude fiber (Fig. 4f) were significantly affected by MNBO stage (*p* < 0.05), and there was no obvious difference in starch (Fig. 4b). The contents of crude ash, vitamin C and crude fiber in the O_20_A treatment increased by 4.20–39.62%, 3.70–39.29% and 3.44–39.29%, respectively, compared to those in the other MNBO stage treatments. MNBO applied at the reproductive stage and grouting maturation stage of spring maize improved the fat content, with significant differences between O_20_V, O_20_A and CK (p < 0.05).

The effects of DO concentration on crude ash, fat and vitamin C of spring maize were significant (*p* < 0.05), but the effect on the starch content was not significant (*p* > 0.05). No significant difference in vitamin C content was observed among the O_10_A, O_30_A and CK treatments, but an obvious elevation of 28.11% was observed in O_20_A compared to CK (*p* < 0.05). The vitamin C content of maize in O_20_A was the highest and was significantly different from those in O_10_A and O_30_A, with increases of 15.97% and 28.08%. MNBO application decreased the crude protein content of the maize kernels. The fat content in maize grain decreased with increasing oxygen concentration, and LSD variance analysis showed no significant difference between O_10_A and CK. However, comparing to CK, the fat content in the maize grain decreased by 5.14% and 24.95% in O_20_A and O_30_A. In O_10_A and O_20_A, the formation of crude ash in maize grain increased by 8.39% and 18.46%.

## Discussion

In this study, field experiment was conducted in two successive years to investigate the effects of the MNBO stage application and DO concentration on maize growth, yield and nutritional quality. The results showed that MNBO under an appropriate DO concentration promoted the growth of maize roots and increased the yield, IWUE and nutritional quality. Similar results have been reported when oxygation was applied to other crops^7,15,34^. Soil moisture and air play contradictory roles when providing water to crops under traditional irrigation methods. Soil moisture removes air from soil pores and reduces the oxygen content of the soil^35^. Moreover, forced ventilation into the soil when the soil moisture is low will exacerbate the decline in soil moisture^20^. Oxygation, which transports the water and air that crops need to the root area, is an expansion of traditional irrigation methods^36^. The soil permeability rate is improved while ensuring soil moisture and improving the oxygen environment in the root zone^37^. The increased soil gas permeability promotes root growth and lateral root formation^38,39^, enhances leaf photosynthesis and root respiration^40^, and relieves the adverse effects of environmental factor stress on crops^41^. Overall, MNBO application improved crop growth, leading to increased yield, WUE and quality^15,34,37,42^.

**Figure 5:**
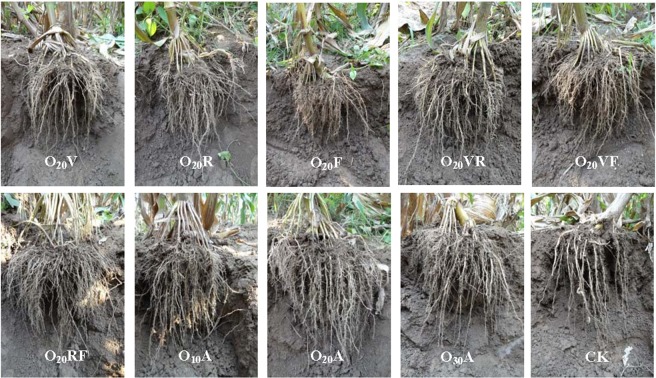
Maize root phonograph taken *in-situ* of different treatments on 100 days after sowing in 2013.

The results indicated that maize roots, yield, WUE, and partial nutrition indicators (crude ash, vitamin C, and crude fiber) under MNBO were superior to those under CK. O_20_A produced more maize grain than did MNBO at each pair of growth stages (O_20_VR, O_20_VF and O_20_RF) and MNBO application at single growth stage (O_20_V, O_20_F, O_20_R). Maize is considered an “intertilled crop”, which means that maize demands substantial amounts of oxygen during the growth period. MNBO applied at the vegetative stages guaranteed the germination of maize seeds after sowing, affecting roots, leaves, and stem node differentiation. MNBO applied at the reproductive stages ensured a vigorous rhizome leaf growth and anthesis-silking interval differentiation and development, which could maintain the kernel emergence and rapid enrichment. It was indicated that the oxygen demand of maize would be better satisfied with MNBO application during the growth period rather than at other stages. Figure 5 shows the morphology of the maize roots on day 100 after sowing. The figure indicates that the longer the MNBO duration was, the greater the growth and development of spring maize roots. Comparison of the O_20_V, O_20_R, O_20_F and CK treatments indicated that the maize root system in O_20_VR, O_20_VF, O_20_RF and O_20_A exhibited characteristics of well-developed surface roots and prosperous lateral roots, exhibiting a multidirectional root system. In addition, oxygation increased the soil microbial abundance and soil enzyme activity^16,43,44^. The decomposition of organic matter in the soil was accelerated and the effectiveness of soil fertility was improved^45^. MNBO applied at different growth stages of maize ensured sufficient nutrient contents throughout the growth period, which promoted the transformation and absorption of nutrients and significantly enhanced the yield and quality of maize.

The effects of DO concentration in irrigation water on maize root growth, yield and nutritional quality were investigated considering three DO concentrations during the growth period. The O_10_A and O_20_A treatments developed shallow roots and exuberant lateral roots compared with those in O_30_A, which grew single roots with fewer lateral roots. The RDW of maize increased as DO concentration increased. However, the maize yield, crude ash, starch and vitamin C trended to increase initially and then decreased with increasing DO concentration. Under 10 mg/L DO, the optimal oxygen content required for maize could not be met, and 30 mg/L DO provided excess oxygen to the roots, causing physiological damage to plant and inhibiting leaf area expansion, relative leaf growth rate and crop growth rate^21,46^. These changes caused a spindling phenomenon in maize roots. Although a higher RDW was obtained, the root tissues were damaged, which affected the absorption of water and nutrients, resulting in crop failure^21^. Because of trial limits, the experiment studied only MNBO at different crop growth stages in the same DO concentration. Further exploration of whether the same DO concentration should be used at different growth stages of maize is necessary. The results demonstrated that MNBO under a high DO concentration adversely affected crop yield^47^. The specific threshold should be further refined according to crop variety and planting conditions.

## Conclusions

The present study was conducted over a two-year period to evaluate the response of maize to MNBO considering MNBO stage and DO concentration. The results demonstrated that MNBO applied during the growth period under appropriate DO concentration significantly affected maize roots, yield, WUE and nutritional quality. The highest yield was obtained in O_20_A and was 11.66% greater than that in CK. Crude ash, starch and vitamin C were also improved. However, excess oxygen adversely affected maize growth that O_30_A treatment decreased the maize yield by 10.97% in 2013 in comparison to CK. The results suggest that MNBO application at 20 mg/L DO during the growth period of spring maize was appropriate.

## Material and Methods

### Site description

The experiment was performed over a two-year period (2013–2015) in two adjacent and homogeneous fields at Beijing Tongzhou experimental station of the China Agricultural University (39°42′N, 116°42′E). The local climate is a continental warm temperate, semi-humid monsoon climate, affected by the winter and summer monsoons, exhibiting the characteristics of hot and rainy summers, cold and dry winters, and a short spring and autumn. The annual average temperature and rainfall are 11.3 °C and 620 mm, respectively. The maize plants were grown in clay loam soils. The experimental site is saline land in Beijing, China, with the characteristics of high soil bulk density and poor soil aeration. Soil characteristics for the 0–100 cm layers are provided in Table 3. The spring maize (cv. “Nongda 86”) crops were sown on May 4^th^ and May 14^th^ and harvested on Sep. 20^th^ and Sep. 28^th^ in 2013 and 2014, respectively.

**Table 3:** Selected physicochemical properties of the soil at depths 0–10, 10–20, 20–40, 40–60 and 60–80 cm prior to the start of the experiment in 2013 and 2014.

Layer	pH		Organic matter		Total N		Total P		Total K		Available N		Available P		Available K	
	2013	2014	2013	2014	2013	2014	2013	2014	2013	2014	2013	2014	2013	2014	2013	2014
0–10 cm	7.3	7.2	300.0	296.7	0.23	0.22	595.5	639.8	11.9	11.0	0.067	0.063	28.9	32.0	183.5	168.9
10–20 cm	7.4	7.4	275.2	282.7	0.20	0.20	579.0	661.1	11.9	12.2	0.058	0.059	31.6	33.9	178.4	187.8
20–40 cm	7.6	7.6	260.8	256.0	0.16	0.16	354.2	359.3	21.0	20.9	0.041	0.037	25.5	26.2	219.7	216.3
40–60 cm	7.6	7.6	262.7	257.3	0.21	0.16	351.0	374.2	12.8	12.9	0.049	0.042	24.3	25.6	197.8	204.5
60–80 cm	7.5	7.5	285.2	306.0	0.20	0.25	346.8	371.2	14.6	13.3	0.056	0.071	25.4	24.8	199.7	205.6

Notes: Organic matter in g/kg. Total N and available N in %. Total P, total K, available P, available K in mg/kg.

### Experimental design and treatment

Field experiment was arranged as a split-plot design arranged in randomized blocks for two consecutive years (2013 and 2014). The experiment consisted of two factors including DO concentration in irrigation water and MNBO stage. In the spring maize growth period, the emergence stage, trefoil stage, jointing stage and small trumpet period are considered the vegetative stage (V); the big trumpet period, tasseling stage, and spinning stage comprise the reproductive stage (R); and the postulation stage, milk-ripe stage, dough stage and full ripe stage are considered the filling and ripening stage (F) of maize. There were seven treatments with MNBO application at individual and paired growth stages under 20 mg/L DO: O_20_V, O_20_R, O_20_F, O_20_VR (MNBO at V and R), O_20_VF (MNBO at V and F), O_20_RF (MNBO at R and F), O_20_A (MNBO at the whole growth stage). There were four treatments with MNBO applied throughout the growth period under different DO concentrations: O_10_A (10 mg/L), O_20_A (20 mg/L), O_30_A (30 mg/L) and CK (control group, nonoxygenated water with a DO of 5 mg/L). There were a total of ten treatments.

Each replication occupied 126 m^2^ (30 m × 4.2 m) consisting of six rows. The maize plants were cultivated in wide/narrow planting rows with a wide-row spacing of 80 cm and narrow-row spacing of 60 cm. The subsurface drip tape was cylindrical pipelines (16 mm in diameter) with a flow rate of 2.6 L/h, a wall thickness of 0.4 mm, and an emitter spacing of 30 cm. The drip tapes were parallel to the maize planting ridges and buried 10–15 cm beneath the surface. The initial flow rate of the drip tape was tested before the experiment, and no significant variation during initial flow was observed along the laterals.

The layout of micro/nanobubbles generator connection, SDI pipeline and buried drip laterals is shown in Fig. S1. The irrigation water pumped from groundwater aquifer was oxygenated by MNB generator with initial 4–5 mg/L DO. A fiber optic trace oxygen meter (Fibox 4 Trace, PreSens, Germany) was used to measure the DO during generator operation time. The device collected DO concentration data every five seconds and fed it back to the display screen. The irrigation water was continuously oxygenated until the setting DO concentration was detected by the device. The MNB water was transported to crop root zone through totally enclosed drip pipe network system and DO in the irrigation water based on *in-situ* measurement is shown in Fig. S2. The method used for generating MNBs of the generator was pressurized gas-liquid mixing. The mean size and numbers of bubbles in water were determined via Nano-Particle Tracking Analysis (NanoSight NS300, Marlern, UK). The bubbles suspension mean size was between 320.08 and 1215.55 nm. The bubble concentration was 3.27 × 10^8^ particles/ml. The zeta potential of the MNB surface was −13.7 mV.

### Irrigation and fertilization regime

A meteorological station was set up at the experimental site to continuously observe the meteorological conditions during the study. During the experimental period, the precipitation during the maize growing was 203.7 mm and 348.8 mm in 2013 and 2014, respectively, which mainly occurred in July and August. The soil moisture content was monitored via TDR (time domain reflectometry) after sowing, which provided soil moisture (%) at soil depths of 10, 20, 40 and 60 cm. The soil moisture content was measured once per week and additionally after irrigation and rainfall. The irrigation quota in 2013 and 2014 were 286.5 mm and 270.9 mm. Figure S3 shows the daily precipitation and irrigation quotas over the two-year trial period. Each treatment was equipped with a water meter to accurately control irrigation quantity. The amount of fertilizer for the ten treatments was identical. All the agronomic cultivation management measures including the irrigation quota and fertilizer amount were the same throughout the experiments except for experimental factors.

### Measurements

Fifteen typical maize plants were sampled at the beginning of experiment and tagged to measure the maize height and stem diameter every 15 days from the beginning of the experiment to the end. The maize height was measured from the plant base to the last opened leaf and the stem diameter was determined with a Vernier caliper 10 cm above the plant base. The roots were excavated with a 0.10 m diameter soil auger 100 days after sowing and each treatment was randomly sampled with five representative maize plants. Soil cores were extracted at six depths (0–10, 10–20, 20–30, 30–40, 40–50 and 50–60 cm) and mixed from three points (¼ row spacing, ½ row spacing and ½ line spacing). The samples were brought to the laboratory for analysis of RDW, RLD and RSA.

The maize yield was estimated by harvesting fifteen tagged maize plants at the end of the physiological maturity. The samples were dried in an open environment, and the yield was determined after threshing. Total grains from a single maize were weighted. The number of maize on the tagged plants was counted. *IWUE*, *RUE* and WUE were calculated using the following formulas:

1 $$IWUE=Y/I$$

2 $$RUE=Y/R$$

3 $$WUE=Y/W$$

where *Y* is the maize yield (kg/ha), *I* is the irrigation quota (m^3^), *R* is the rainfall per hectare (m^3^), and *W* is the consumed water amounts per hectare (m^3^).

Five maize per treatment were sampled for the nutritional quality measurements. The threshed maize kernels were evenly mixed, and each nutritional quality test sample weighed 200 g. Crude fiber was measured via the intermediate filtration method. The fat content was determined via the Soxhlet extractor method. The starch, crude protein, and crude ash were determined via near-infrared absorption, the Kjeldahl method, and combustion at 550 °C, respectively. The vitamin C content was measured using the 2,6-dichloro-indophenol titration method.

### Data analysis

All data are presented as the mean ± standard deviation. ANOVA was performed using the SPSS 22.0 software package. The least significant difference (LSD) test at a *p* value of 0.05 was used to separate treatment means exhibiting significant differences.

## Supplementary information

Supplementary materials
