# Supplementary material for: Synergistic improvement in spring maize yield and quality with micro/nanobubbles water oxygation
Source: Sci Rep. 2019 Mar 26;9:5226. doi: 10.1038/s41598-019-41617-z (PMC6435734; doi:10.1038/s41598-019-41617-z)
Supplement: Supplementary file 1 — Supplementary materials [file 41598_2019_41617_MOESM1_ESM.docx]

**Synergistic improvement in spring maize yield and quality with micro/nanobubbles water oxygation**

**Yunpeng Zhou^1^, Yunkai Li^1^* Xiujuan Liu^2^, Keyuan Wang^1^, Tahir Muhammad^1^**

^1^ College of Water Resources and Civil Engineering, China Agricultural University, Beijing 100083, China

^2^ Fujian Provincial Investigation, Design & Research Institute of Water Conservancy & Hydropower, Fuzhou 350001, China

**Correspondence to*: liyunkai@126.com**Captions for Figures in the Supplementary materials**

Figure S1. Layout of micro/nanobubbles generator connection, subsurface drip irrigation pipeline and buried drip laterals.

Figure S2. Dissolve oxygen (DO) in the irrigation water based on in-situ measurement.

Figure S3. Daily rainfall (mm) and irrigation amounts (mm) during the maize growth seasons in 2013 and 2014.

**Figure S1. Layout of micro/nanobubbles generator connection, subsurface drip irrigation pipeline and buried drip laterals.**


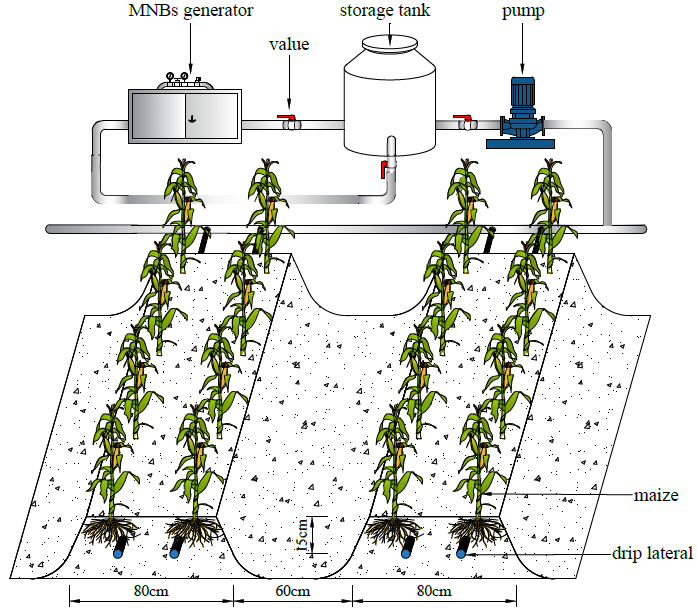


**Figure S2.** Dissolve oxygen (DO) in the irrigation water based on in-situ measurement.

| **A** | **B** |
| --- | --- |
| **C** | A. DO changes with oxygenation time.  B. DO changes with preservation time.  C. DO changes along with the dripline. |

The DO of non-oxygenated water was about 4-5 mg/L. An Oxygen Meter (Fibox 4 Trace, PreSens, Germany) was introduced to measure the DO during oxygenation and along the dripline. The change of DO from 0-60 min in the process of micro/nanobubbles generator operation was shown in Fig.1A. It was indicated that the DO exhibited an initial increase with a rate of 0.0014 mg L^-1^/s, and stabilized after 50 minutes. The highest DO of 8.2 mg/L in water was observed when using air as gas resource. It indicated the DO of micro-nanobubbles water could still maintain a high level of 7.80 mg/L after 200h, with trend of 5% decrease compared to initial status and an increase of 95% compared to non-oxygenated water. There was no noticeable variation of DO in micro-nanobubbles water along with 60 m dripline. The rang of DO was less than 5%. Micro-nanobubbles water was stable in the process of transport, and the change of DO along the dripline was not obvious, that is, in the actual irrigation process, the DO distribution in the root zone of crops was relatively uniform.

**Figure S3. Daily Rainfall (mm) and irrigation amounts (mm) during the maize growth seasons in 2013 and 2014.**

**A. 2013**

**B. 2014**
